# The relationship between plasma favipiravir concentrations and clinical outcomes in COVID-19

**DOI:** 10.1183/23120541.01560-2025

**Published:** 2026-07-27

**Authors:** Rebecca E. Wawman, Pallav L. Shah, Marta Boffito, James Tonkin, Francesca Conway, Anand Tana, Breno R. Santos, Beatriz Grinsztejn, Brenda Crabtree Ramírez, Henry Pertinez, Andrew Owen, Paul Curley, Usman Arshad, Helen Cox, Mark R. Johnson, Anton Pozniak, Michael Pelly, Christopher M. Orton, Pankaj K. Bhavsar

**Affiliations:** 1 National Heart and Lung Institute, Imperial College London, London, UK; 2 Department of Respiratory Medicine, Royal Brompton Hospital, London, UK; 3 Chelsea and Westminster NHS Foundation Trust, London, UK; 4 Departamento de Infectología, Hospital Nossa Senhora da Conceição–Grupo Hospitalar Conceição, Porto Alegre, Brazil; 5 Instituto Nacional de Infectologia Evandro Chagas, Rio de Janeiro, Brazil; 6 Departamento de Infectología, Instituto Nacional de Ciencias Médicas y Nutrición, Salvador Zubirán, Mexico City, Mexico; 7 Department of Pharmacology and Therapeutics, Institute of Systems, Molecular and Integrative Biology, University of Liverpool, Liverpool, UK; 8 Department of Clinical Research, London School of Hygiene and Tropical Medicine, London, UK

## Abstract

**Background:**

Favipiravir has shown efficacy against SARS-CoV-2 in patients <60 years old, but data linking plasma concentrations to clinical outcomes are limited. This study investigated whether favipiravir plasma concentrations influence clinical efficacy and outcomes in patients hospitalised with COVID-19. The main research question was, How can antiviral dosing strategies be optimised to improve pandemic preparedness and treatment efficacy?

**Methods:**

Adult participants were drawn from the PIONEER trial, in which patients received oral favipiravir (1800 mg twice daily for 1 day, then 800 mg twice daily for 9 days) plus standard care. This analysis included patients with confirmed COVID-19 and ≥75% study adherence. Samples were collected between days 5 and 10 post-treatment initiation. The primary outcome was time to clinical improvement. Secondary outcomes included achievement of clinical improvement and mortality risk.

**Results:**

Out of 140 patients (50% male; mean±sd age 59.5±14.8 years), target plasma concentrations were reached in 29 (21%). Mean time to improvement was 7.7±5.9 days in target achievers *versus* 9.1±7.2 days in non-achievers (p=0.26). The target was more often achieved in female (34%) than male (7%) participants (p=0.0002). Plasma concentration inversely correlated with body mass index (r= –0.4, p<0.0001), and with lower body mass index in achievers (26.0±5.1 kg·m^−2^ *versus* 30.5±6.9 kg·m^−2^, p=0.003). Alkaline phosphatase and alanine aminotransferase levels were also lower in achievers (p=0.004 and p=0.02, respectively).

**Conclusion:**

Most patients did not reach target favipiravir levels. Concentrations were influenced by sex, body mass index and liver function, confirming the need for pharmacokinetically guided dosing and therapeutic monitoring to optimise antiviral efficacy in future pandemic responses.

## Shareable abstract

**Plasma favipiravir concentrations vary with sex, BMI and liver function. Concentrations in most patients fail to reach therapeutic levels, highlighting the need for personalised, pharmacokinetically guided dosing to optimise antiviral efficacy in future pandemics.** https://bit.ly/4a6PvEa

## Introduction

Favipiravir, an antiviral medication, has exhibited efficacy against SARS-CoV-2 in laboratory and animal studies [1–3], and has a well-established safety profile in treating influenza [4]. Despite this, the PIONEER clinical trial, which assessed the efficacy of favipiravir in treating patients hospitalised with COVID-19, found a beneficial effect on recovery and survival only in patients <60 years old [5]. Although significant variability in blood favipiravir concentrations have been reported [6], the optimal concentration required for effective COVID-19 therapy may exceed doses studied during the pandemic [7]. There remains a lack of published data linking blood favipiravir concentrations to therapeutic efficacy.

Favipiravir is absorbed orally, with a bioavailability of approximately 94% and peak plasma concentrations reached within 2 h [8]. It has a moderate volume of distribution and a plasma-protein binding rate of approximately 54% [8]. The metabolism of favipiravir primarily occurs hepatically *via* aldehyde oxidase [9]. Excreted *via* the kidneys, the half-life of favipiravir is 2–5 h; renal impairment can prolong this [9]. Favipiravir serves as a prodrug that, upon administration, undergoes intracellular phosphorylation to produce the favipiravir–ribofuranosyl-5′-triphosphate (RTP) active metabolite. In the phosphoribosylated state, favipiravir specifically acts on the active site of RNA-dependent RNA polymerase (RdRp), a critical enzyme for viral genome replication, thereby impeding viral RNA polymerase activity and viral replication [10]. Research by Shannon *et al*. [11] indicates that the RdRp complex of SARS-CoV-2 is significantly more active than that of other known viruses. Determining the optimal dose of favipiravir for COVID-19 treatment remains challenging owing to the limited preclinical and *in vitro* data available. Preclinical studies indicate that the effective favipiravir plasma concentration required to inhibit the Ebola virus is significantly greater than that needed for influenza [12, 13]. In the JIKI trial, however, which assessed favipiravir efficacy in patients with Ebola virus, expected target concentrations of favipiravir were not reached, even with elevated dosing [14].

This study aimed to investigate the relationship between plasma favipiravir concentrations and clinical outcomes in patients with COVID-19. We hypothesised that achieving higher plasma concentrations of favipiravir will correlate with improved clinical outcomes.

## Materials and methods

### Study participants

Blood samples and clinical data were obtained from adult patients hospitalised with COVID-19 who were randomised 1:1 to receive oral favipiravir (1800 mg twice daily for 1 day, then 800 mg twice daily for 9 days) plus standard care, or standard care alone, as part of the PIONEER trial between May 2020 and May 2021. All patients included in these further analyses had confirmed COVID-19 and favipiravir adherence >75%. Changes in clinical status were assessed on a seven-category ordinal scale (supplementary table S1), with the primary outcome measured as the time from randomisation to recovery of two or more points, or hospital discharge. Recruitment for the PIONEER trial was completed during the early waves of the COVID-19 pandemic, when vaccines were not yet widely available, and the study population therefore largely reflects unvaccinated hospitalised patients.

### Pharmacokinetic and pharmacodynamic analysis

Plasma was isolated from patients at a single time point 5–10 days post-initiation of favipiravir. Plasma concentrations of favipiravir were then quantified using a validated liquid chromatography coupled with tandem mass spectrometry method, as previously described by Curley *et al.* [15]. A target favipiravir concentration of 25 000 ng·mL^−1^ (159 μM) was chosen for comparison with observed exposures, based on the *in vitro* concentration needed to achieve 90% of its maximal effect (EC_90_) against SARS-CoV-2 in a Vero E6 cell-based assay [7, 16]. Plasma sampling was performed at a single time point between 5 and 10 days after treatment initiation to capture favipiravir exposure following several days of sustained twice-daily dosing during the maintenance phase of therapy. Favipiravir exhibits time-dependent and nonlinear pharmacokinetics; therefore, this mid-treatment window was selected to reflect on-treatment exposure rather than early concentrations immediately following the loading dose.

### Statistical analysis

The chi-squared test was used to assess variance in the frequency of categorical data, with data presented as absolute number (%), and t-tests were used to assess variance in means, with data presented as mean±sd. Survival data were analysed using the Kaplan–Meier method to estimate survival probabilities over time. The overall survival curves were compared using a Cox proportional hazards model. To evaluate pairwise differences between groups, the log-rank test was applied. p-values obtained from these comparisons were adjusted for multiple testing using the Benjamini–Hochberg procedure to control for false discovery. All statistical analyses were performed in the R statistical environment (version 4.4.0; www.r-project.org), with p<0.05 considered statistically significant.

### Sample size consideration

This study was a *post hoc* analysis from the PIONEER trial. The sample size was therefore pragmatic and determined by the number of participants randomised to favipiravir with 1) confirmed COVID-19, 2) available plasma samples collected 5–10 days after treatment initiation, and 3) ≥75% adherence. A formal *a priori* sample size calculation was not performed for this secondary analysis.

## Results

### Pharmacokinetics and pharmacodynamics of favipiravir

Plasma concentrations of favipiravir were measured in 140 patients at a single time point 5–10 days post-initiation of favipiravir (supplementary figure S1). Patient characteristics, including comorbidities, time from symptom onset to treatment initiation and baseline disease severity, are described in table 1. 29 patients (21%) reached target plasma favipiravir concentrations of >25 000 ng·mL^−1^ (mean±sd concentration of 44 561.8±30 367.7 ng·mL^−1^), and 111 (79%) did not (9017.9±7225.4 ng·mL^−1^; table 1). Time to clinical improvement was 7.7±5.9 days among target achievers and 9.1±7.2 days among non-achievers (p=0.26; table 1). Clinical improvement occurred in 97% of patients who met the target concentration and in 83% of patients who did not meet the target concentration (p=0.12; table 1). Adverse events were reported in 14 patients who met target concentrations (48%) and in 60 patients who did not (54%, p=0.73; table 1). Of the patients who met the target concentration, 83% (24 out of 29) were female, compared with 41% (46 out of 111) of the patients who did not (p=0.0002; table 1). The mean age of patients who met the target concentration was 62.9±13.7 years, and the mean age of those who did not meet the target concentration was 58.6±14.9 years (p=0.15; table 1). Patients ≥60 years old constituted 62% of those who met the target concentration, and 53% of those who did not (p=0.52; table 1). Of the male patients with available plasma samples, 7% met the target concentration, compared to 34% of female patients (p=0.0002). Female patients had a significantly higher mean plasma favipiravir concentration of 22 875.8±26 470.0 ng·mL^−1^ *versus* 9687.4±8961.9 ng·mL^−1^ in male patients (p=0.0001; table 2).

**TABLE 1:** Characteristics of patients who achieved target plasma favipiravir concentrations compared to those who did not

	Target concentration achieved		
	Yes	No	p-value
**Total participants, n**	29	111	
**Time to clinical improvement, days**			
Mean±sd	7.7±5.9	9.1±7.2	0.26
Range	1–18	1–28	
**Clinical improvement achieved**			
Yes	28 (97)	92 (83)	0.12
No	1 (3)	19 (17)	
**Adverse event occurred**			
Yes	14 (48)	60 (54)	0.73
No	15 (52)	51 (46)	
**Baseline ordinal score**			
Mean±sd	3.7±0.5	3.9±0.5	0.006
Range	3–4	3–5	
**Time from symptom onset to treatment initiation, days**			
Mean±sd	9.3±2.9	8.4±3.5	0.18
Range	3–15	1–23	
**Gender**			
Male	5 (17)	65 (59)	0.0002
Female	24 (83)	46 (41)	
**Age, years**			
Mean±sd	62.9±13.7	58.6±14.9	0.15
<60	11 (38)	52 (47)	0.52
≥60	18 (62)	59 (53)	
**BMI, kg·m^−2^** ^#^			
Mean±sd	26.0±5.1	30.5±6.9	0.006
Range	14.7–36.1	16.0–57.0	
**BMI category, kg·m^−2^**			
Underweight (BMI <18.5)	2 (8)	2 (2)	0.04
Healthy weight (BMI 18.5–24.9)	10 (38)	16 (17)	
Overweight (BMI 25–29.9)	8 (31)	32 (34)	
Obesity (BMI 30–39.9)	6 (23)	36 (38)	
Severe obesity (BMI >40)	0 (0)	9 (9)	
**Creatinine, μmol·L^−1^**			
Mean±sd	93.6±52.9	91.5±35.9	0.84
Range	53.0–342.1	42.4–350.0	
**Potassium, mmol·L^−1^**			
Mean±sd	4.1±0.4	4.3±0.5	0.08
Range	3.6–5.1	3.2–5.9	
**Urea, mmol·L^−1^**			
Mean±sd	11.2±6.7	11.2±6.1	0.99
Range	3.5–38.9	2.2–36.3	
**eGFR, mL·min^−1^·1.73 m^−2^**			
Mean±sd	69.4±21.8	77.8±23.6	0.08
Range	12.0–107.0	15.9–142.7	
**Renal comorbidity**			
Yes	1 (3)	8 (7)	0.76
No	28 (97)	103 (93)	
**ALP, U·L^−1^** ^¶^			
Mean±sd	72.0±24.6	93.1±52.9	0.004
Range	34.0–130.0	29.0–359.0	
**ALT, U·L^−1^** ^¶^			
Mean±sd	33.2±21.0	45.4±32.8	0.02
Range	6.0–90.0	7.0–186.0	
**Hepatic comorbidity**			
Yes	2 (7)	10 (9)	1.00
No	27 (93)	101 (91)	
**Comorbidity**			
Yes	23 (79)	82 (74)	0.55
No	6 (21)	29 (26)	
Mean±sd per patient	1.3±1.0	1.5±1.4	0.40
Range	0–3	0–5	

Data presented as n (%), unless otherwise stated. The chi-squared test was used to assess variance in frequencies of categorical data and the t-test was used to assess variance in means between favipiravir target plasma concentration achievers and non-achievers. ^#^: BMI data available for n=121 patients; ^¶^: to convert U·L^−1^ to µkat·L^−1^, multiply by 0.0167.

**TABLE 2:** Plasma favipiravir concentrations in male compared to female patients

	Male	Female	p-value
**Total participants, n**	70	70	
**Target concentration of favipiravir met, n (%)**			
Yes	5 (7)	24 (34)	0.0002
No	65 (93)	46 (66)	
**Plasma concentration of favipiravir (ng·mL^−1^)**			
Mean±sd	9687.4±8961.9	22 875.8±26 470.0	0.0001
Range	6.6–34 322.5	3.01–144 745.0	

The chi-squared test was used to assess variance in frequencies of categorical data and the t-test was used to assess variance in means.

**FIGURE 1:**
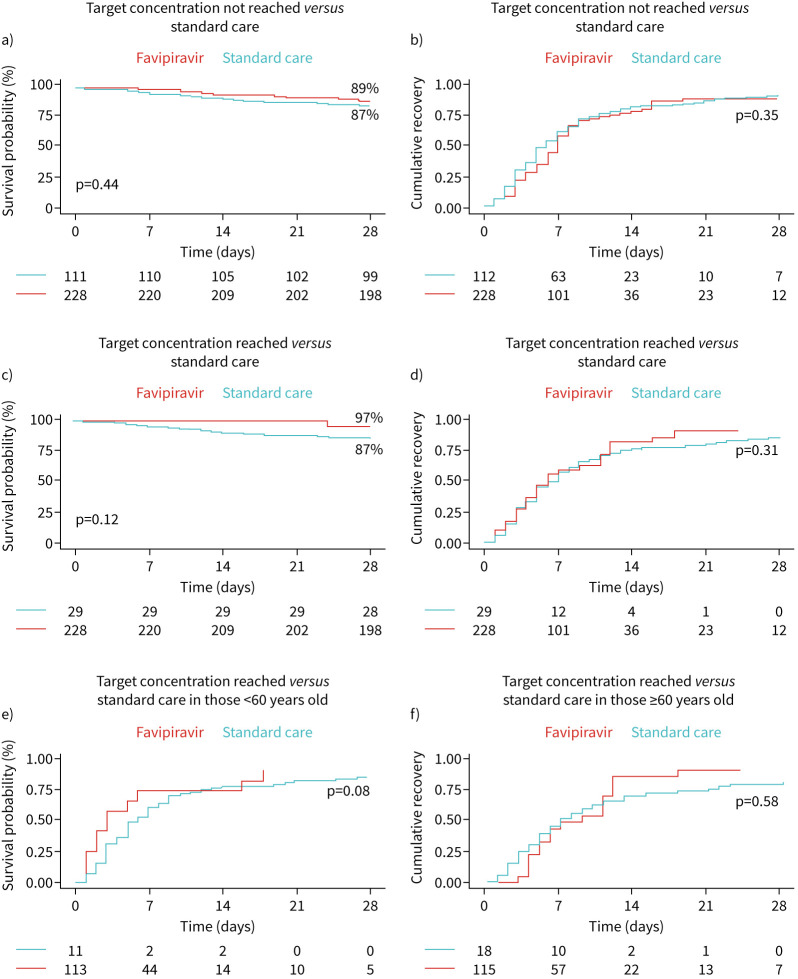
28-day survival and cumulative recovery of patients who met and did not meet target plasma favipiravir concentrations relative to standard care. Kaplan–Meier curves were used to visualise patient mortality and cumulative recovery over 28 days for a, b) favipiravir recipients who did not meet target plasma drug concentrations (n=111 and n=112, respectively) compared to standard care recipients (n=228); c, d) favipiravir recipients who did reach target plasma drug concentrations (n=29) compared to standard care recipients (n=228); e) patients <60 years old who met target plasma favipiravir concentrations (n=11) compared to patients <60 years old who received standard care (n=113); and f) patients ≥60 years old who met target plasma favipiravir concentrations (n=18) compared to patients ≥60 years old who received standard care (n=115). Comparison of survival curves was made using a Cox proportional hazards model.

Patients who did not achieve the target concentration exhibited no significant difference in 28-day survival (hazard ratio (HR) 1.30, 95% confidence interval (CI) 1.95–12.59, n=339, p=0.44) or time to clinical improvement (HR 1.12, 95% CI 2.39–4.20, n=340, p=0.35) relative to patients who received standard care (figure 1a, b). Clinical improvement was achieved in 97% of patients who reached the target concentration (28 out of 29 patients), compared with 81% of standard care recipients (185 out of 238, p=0.07; table 3). Patient 28-day survival was 97% for those who reached the target concentration, relative to 87% for standard care recipients (HR 1.44, 95% CI 1.78 to –2.75, n=257, p=0.12; figure 1c). There was no difference in time to clinical improvement between patients who reached the target concentration and standard care recipients (HR 0.82, 95 CI 1.73–3.39, n=257, p=0.31; figure 1d). When further stratified by age, patients <60 years old who reached the target concentration had a nonsignificant numerical reduction in time to clinical improvement when compared to recipients of standard care (HR 0.64, 95% CI 1.40–3.42, n=124, p=0.08; figure 1e). No difference was seen in the time to clinical improvement in patients ≥60 years old who reached target concentrations and recipients of standard care (HR 0.82, 95% CI 1.70 to –2.98, n=133, p=0.58; figure 1f).

**TABLE 3:** Outcomes of patients who met target plasma favipiravir concentrations compared to standard care recipients

	Target concentration achieved		
	Favipiravir (target achieved)	Standard care	p-value
**Total participants, n**	29	228	
**Time to clinical improvement, days**			
Mean±sd	7.7±5.9	8.1±7.2	0.71
Range	1–24	1–28	
**Clinical improvement achieved, n (%)**			
Yes	28 (97)	185 (81)	0.07
No	1 (3)	43 (19)	

The chi-squared test was used to assess variance in frequencies of categorical data and the t-test was used to assess variance in means.

### Body mass index

**FIGURE 2:**
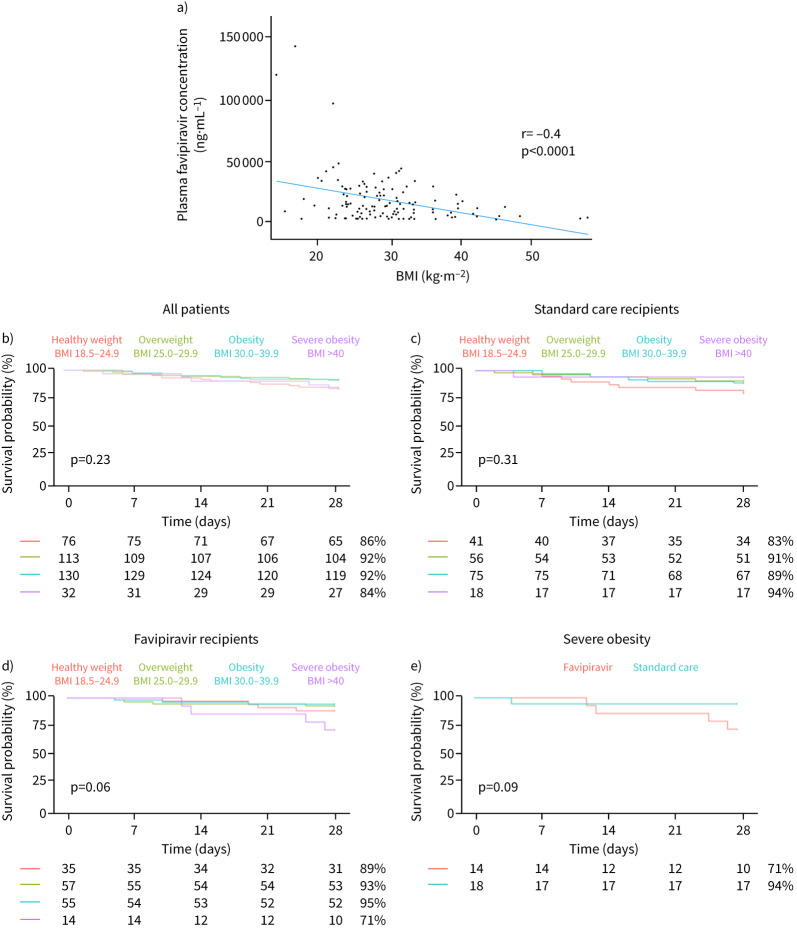
Plasma favipiravir concentrations correlated with patient body mass index (BMI) and 28-day survival of patients by BMI. a) Patient plasma favipiravir concentrations were quantified days 5–10 post-treatment initiation and correlated against patient BMI (in kg·m^−2^) at the time of treatment initiation. Pearson's correlation coefficient was calculated to assess the linear relationship between concentration and BMI. b–e) Kaplan–Meier curves were used to visualise patient mortality over 28 days for all patients with a healthy weight (n=76), overweight (n=113), obesity (n=130) and severe obesity (n=32) (b); standard care recipients with a healthy weight (n=41), overweight (n=56), obesity (n=75) and severe obesity (n=18) (c); favipiravir recipients with a healthy weight (n=35), overweight (n=57), obesity (n=55) and severe obesity (n=14) (d); and patients with severe obesity who received favipiravir treatment (n=14) compared to standard care treatment (n=18) (e). Comparison of survival curves was made using a Cox proportional hazards model.

Body mass index (BMI) data were obtained for 121 of 140 patients in whom plasma samples were collected (86%). Patients who met the target concentration had a mean BMI of 26.0±5.1 kg·m^−2^, which was significantly lower than the BMI of those who did not meet the target concentration (30.5±6.9 kg·m^−2^, p=0.006; table 1). There was an increased proportion of patients with a healthy bodyweight (BMI 18.5–24.9 kg·m^−2^) (n=10, 38%), and a decreased proportion of both obesity (BMI 30.0–39.9 kg·m^−2^) (n=6, 23%) and severe obesity (BMI >40 kg·m^−2^) (n=0, 0%) amongst those who met the target concentration relative to those who did not (healthy weight: n=16, 17%; obesity: n=36, 38%; severe obesity: n=9, 9%; p=0.04; table 1). *Post hoc* pairwise chi-squared tests revealed a significant difference in the proportion of patients who met and did not meet the target concentration in patients of a healthy weight *versus* patients with obesity (p=0.05; supplementary table S2). BMI negatively corelated with favipiravir plasma concentrations (r= −0.4, p<0.0001; figure 2a).

Patient 28-day survival was not significantly associated with BMI in the total population (HR 0.89, 95% CI 0.62–1.27, n=351, p=0.23; figure 2b), in patients receiving standard care alone (HR 0.71, 95% CI 0.45–1.12, n=190, p=0.31; figure 2c) or in patients receiving favipiravir (HR 1.24, 95% CI 0.70–2.17, n=161, p=0.06; figure 2d). *Post hoc* pairwise comparisons of favipiravir recipients revealed significant differences in survival between patients with overweight (BMI 25.0–29.9 kg·m^−2^) and with severe obesity (p=0.03), as well as between patients with obesity and with severe obesity (p=0.01; supplementary table 3). No significant between-weight stratifications were shown in the total population (supplementary table 4) or amongst standard care recipients (supplementary table 5). The 28-day survival of favipiravir recipients with severe obesity (71%) was reduced by 23% relative to the 28-day survival of standard care recipients with severe obesity (94%) (HR 0.18, 95% CI 0.02–1.66, n=32, p=0.09; figure 2e). Treatment with either favipiravir or standard care did not, however, affect the survival of participants with a healthy weight (HR 1.83, 95% CI 0.55–6.09, n=76, p=0.31), with overweight (HR 1.28, 95% CI 0.34–4.77, n=113, p=0.71) or with obesity (HR 1.99, 95% CI 0.53–7.49, n=130, p=0.30) (supplementary figure 2).

### Renal function

Serum potassium, urea and creatinine levels, taken at the time of study-arm allocation (baseline), were used, alongside estimated glomerular filtration rate (eGFR) and patient renal comorbidity, to explore the relationship between renal function and plasma favipiravir concentrations. No significant differences were found between those with plasma favipiravir concentrations above and below 25 000 ng·mL^−1^ in the mean baseline levels of potassium (4.1±0.4 *versus* 4.3±0.53 mmol·L^−1^, p=0.08), urea (11.2±6.7 *versus* 11.2±6.1 mmol·L^−1^, p=0.99) or creatinine (93.6±52.9 *versus* 91.5±35.9 µmol·L^−1^, p=0.84), or between eGFRs (69.4±21.8 *versus* 77.8±23.6 mL·min^−1^·1.73 m^−2^, p=0.08; table 1). Furthermore, no significant difference was found in the proportion of patients presenting with a renal comorbidity amongst those who met (n=1, 3%), and did not meet (n=8, 7%) the target concentration (p=0.76; table 1).

Baseline serum potassium levels negatively corelated with plasma favipiravir concentration (r= −0.2, p=0.04; supplementary figure 3A). Baseline levels of creatinine (r= −0.1, p=0.22; supplementary figure 3B) and urea (r= −0.1, p=0.11; supplementary figure 3C) as well as eGFR (r=0.03, p=0.68; supplementary figure 3D) did not corelate with drug plasma concentration.

### Hepatic function

Patients who achieved the target concentration, compared with patients who did not, demonstrated significantly reduced levels of serum alkaline phosphatase (ALP) (72.0±24.6 *versus* 93.1±52.9 U·L^−1^, p=0.004) and alanine aminotransferase (ALT) (33.2±21.0 *versus* 45.4±32.8 U·L^−1^, p=0.02) (SI conversion factor: to convert U·L^−1^ to µkat·L^−1^, multiply by 0.0167) (table 1). There was no difference in the proportion of patients with a hepatic comorbidity between those who reached (7%) or did not reach (9%) the target concentration (p=0.10; table 1). Baseline serum levels of ALT were negatively corelated with plasma favipiravir concentration (r= −0.2, p=0.04; supplementary figure 4A), whereas baseline ALP levels did not (r= −0.1, p=0.18; supplementary figure 4B).

## Discussion

This study investigated the relationship between plasma favipiravir concentrations and clinical outcomes in patients hospitalised with COVID-19, with a focus on pharmacokinetic variability across clinically relevant subgroups defined by gender, BMI and hepatic or renal function. We observed substantial inter-individual variability in plasma favipiravir concentrations, with only a minority of patients achieving the prespecified target exposure, consistent with previous pharmacokinetic modelling and clinical studies reporting lower-than-target concentrations in treated patients [4, 7, 14]. Gender, BMI and hepatic markers (ALT and ALP) were significant determinants of favipiravir exposure, whereas renal function did not show a meaningful association. Women, individuals with lower BMI and those with more favourable baseline liver function were more likely to reach therapeutic concentrations.

The observed sex-based differences in favipiravir exposure align with established evidence demonstrating sex-related variability in pharmacokinetics and pharmacodynamics across a range of antiviral and other therapeutic agents [17, 18]. These differences likely reflect a combination of biological factors, including body composition, volume of distribution and hepatic metabolism. Sex-specific differences in the expression and activity of drug-metabolising enzymes and transporters, including aldehyde oxidase, have been described and may contribute to variability in favipiravir exposure [19–21]. Our findings extend this literature by highlighting the additional influence of BMI and liver enzyme profiles, particularly ALT and ALP, on plasma favipiravir concentrations.

Body composition is a well-recognised determinant of drug disposition, with obesity associated with an expanded volume of distribution and altered clearance, potentially reducing drug availability at sites of action [22–24]. Given that women generally have lower BMI and reduced lean body mass compared with men [25], differences in body composition may partially explain the higher favipiravir concentrations observed in female patients. Similar sex- and BMI-associated differences in plasma drug concentrations have been reported for other fixed-dose therapies, including antidepressants and antiretrovirals, where women achieved higher exposure despite receiving comparable or lower doses [26–28].

Favipiravir is predominantly metabolised by hepatic aldehyde oxidase [1, 19], and hepatic function therefore represents a key determinant of drug exposure. Obesity-related liver dysfunction may impair metabolic capacity through steatosis and inflammation, reducing drug clearance and increasing toxicity risk [29, 30]. Conversely, chronic low-grade inflammation associated with obesity may upregulate drug-metabolising pathways, potentially accelerating clearance and reducing effective drug concentrations [31–35]. In this context, the observed associations between elevated ALP or ALT and reduced likelihood of achieving target favipiravir concentrations suggest that baseline liver enzyme profiles may serve as clinically relevant markers of favipiravir pharmacokinetics. Elevated ALP may reflect biliary or hepatic stress impairing drug metabolism [36, 37], while lower ALT levels may indicate preserved hepatocellular function supportive of more efficient drug handling [37].

A major strength of our study is the identification of factors that need to be considered when deploying antivirals in outbreak settings. However, some limitations should be considered when interpreting our findings. First, this was a *post hoc* analysis nested within a pragmatic randomised trial, and sample size was constrained by plasma availability and adherence criteria. Only a minority of participants achieved target concentrations, resulting in imbalanced group sizes and limited statistical power for some comparisons. Second, plasma favipiravir concentrations were measured at a single time point between days 5 and 10 after treatment initiation. This sampling window was selected to capture exposure following several days of sustained dosing and to approximate steady-state concentrations, consistent with time-dependent and nonlinear pharmacokinetics of favipiravir [1, 4]. This mid-treatment window reflects the maintenance phase of therapy and was chosen to provide a pragmatic estimate of on-treatment exposure in hospitalised patients. However, single time point sampling does not capture intra-individual variability or peak–trough fluctuations over the course of treatment. In addition, preclinical data indicate that favipiravir exhibits time-dependent pharmacokinetics, with an apparent increase in half-life following repeated dosing, suggesting that plasma concentrations may continue to evolve over time rather than remaining constant [10, 38]. Third, intracellular concentrations of the active metabolite favipiravir RTP were not measured, limiting direct inference regarding antiviral activity at the cellular level [7]. Finally, the potential influence of concurrent medications, illness severity and non-hepatic comorbidities on favipiravir pharmacokinetics could not be fully accounted for.

Despite these limitations, this study provides clinically relevant insights into the determinants of favipiravir exposure in patients hospitalised with COVID-19. Recruitment was completed during the early waves of the COVID-19 pandemic, prior to widespread vaccine availability, and the findings therefore primarily reflect treatment responses in unvaccinated patients. Together, these results emphasise the importance of robust preclinical dose-finding, adaptive pharmacokinetic evaluation and consideration of individual patient characteristics when deploying antivirals in outbreak settings. Precision dosing strategies informed by demographic and biochemical parameters may be necessary to optimise antiviral efficacy and minimise harm in future pandemics.

## Supplementary material

**Please note:** supplementary material is not edited by the Editorial Office, and is uploaded as it has been supplied by the author. Supplementary material 01560-2025.SUPPLEMENT
