# Supplementary material for: The relationship between plasma favipiravir concentrations and clinical outcomes in COVID-19
Source: ERJ Open Res. 2026 Jul 27;12(4):01560-2025. doi: 10.1183/23120541.01560-2025 (PMC13432959; doi:10.1183/23120541.01560-2025)
Supplement: Supplementary file 1 [file 01560-2025.SUPPLEMENT.pdf]

Supplementary Material

Supplementary figures

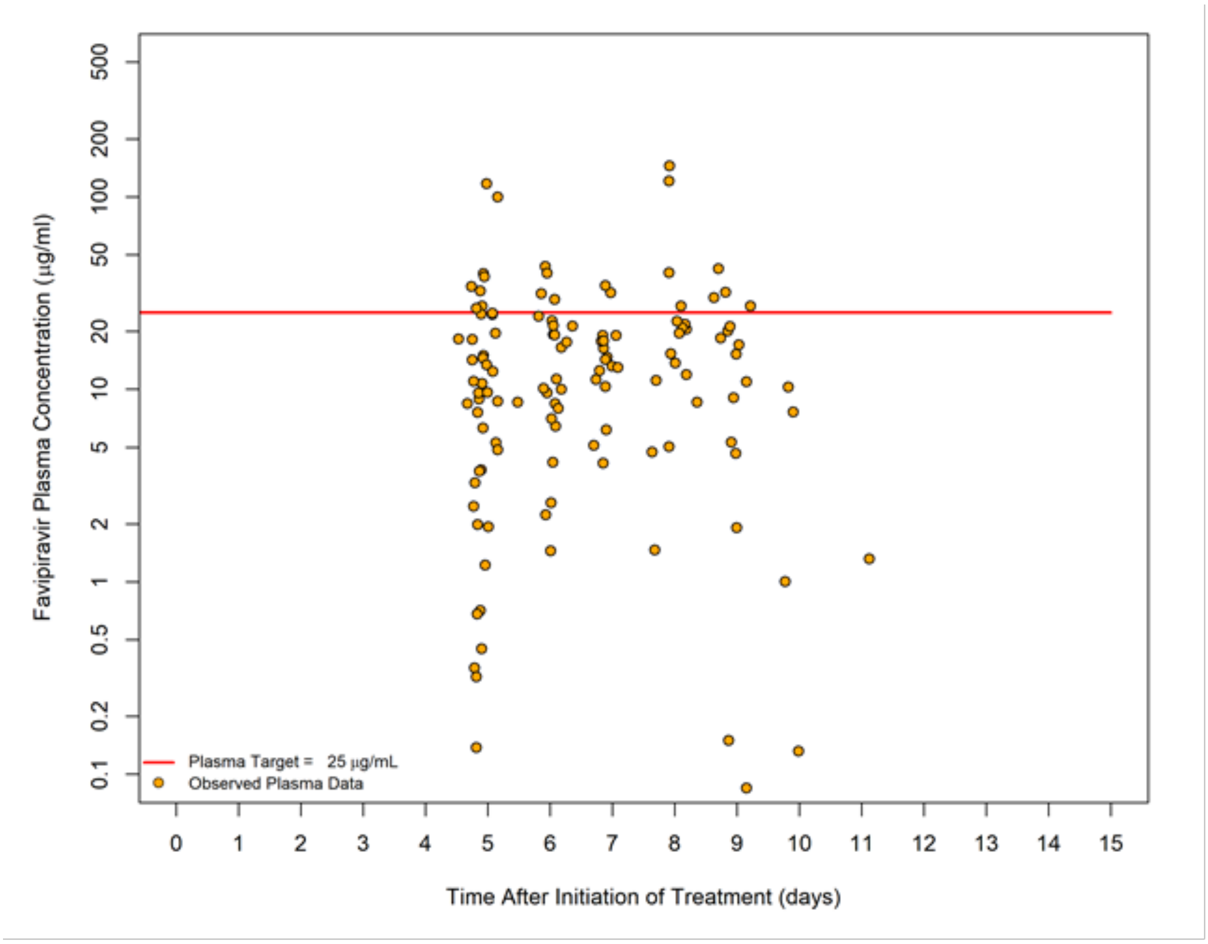

Supplementary Figure 1. Observed Favipiravir plasma concentrations vs. time after initiation of treatment. Individual participant data are presented for the studied dosing regimen of 1800 mg BID on day 1, followed by 800 mg BID maintenance. The red horizontal line represents the proposed plasma concentration target of 25,000ng/mL.

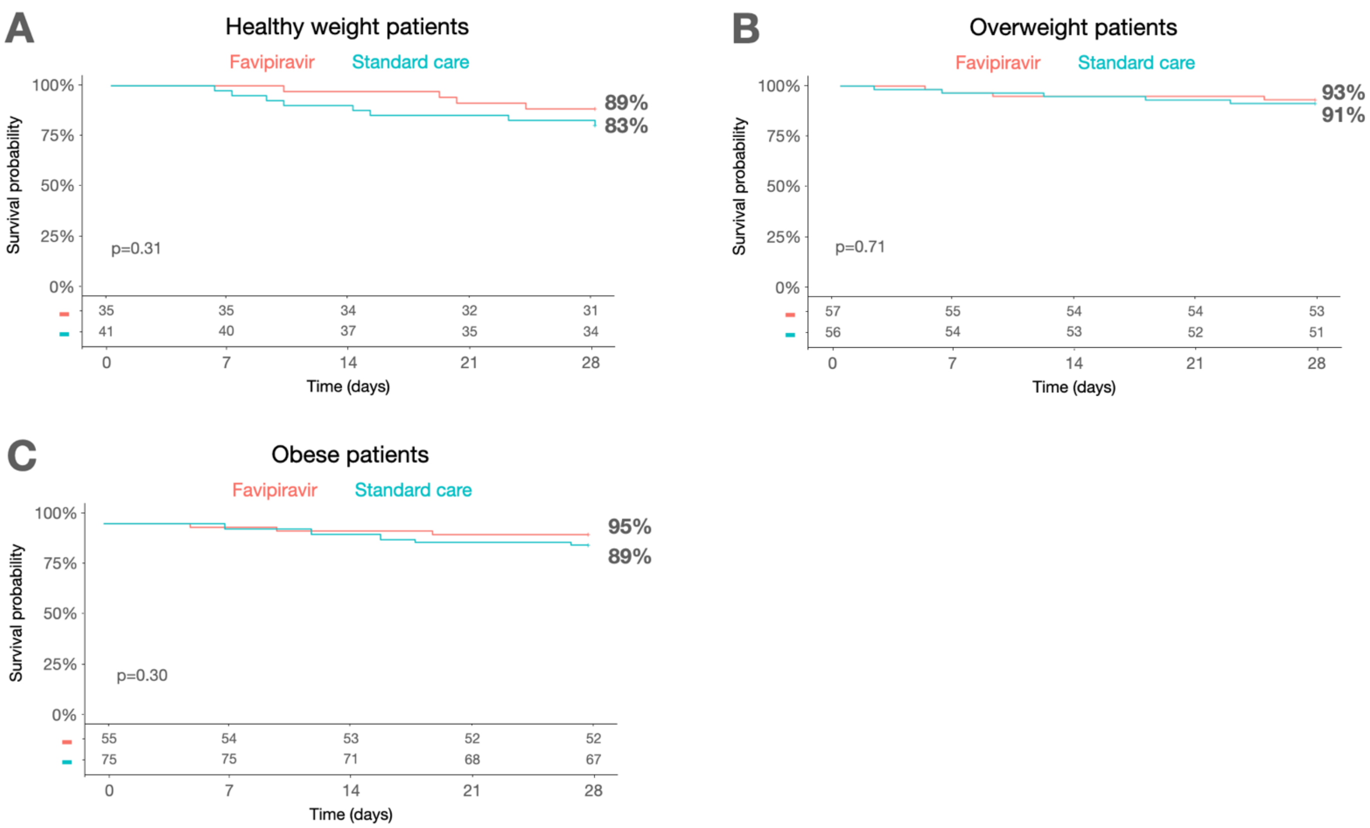

Supplementary Figure 2. 28 day survival of favipiravir and standard care recipients by body mass index. Kaplan-Meier curves were used to visualise patient mortality over 28 days for **A)** Patients of a healthy weight who received favipiravir (n=35) compared to standard care treatment (n=41); **B)** overweight patients who received favipiravir (n=57) compared to standard care treatment (n=56) and **C)** obese patients who received favipiravir (n=55) compared to standard care treatment (n=75). Comparison of survival curves was made using a Cox Proportional Hazards model.

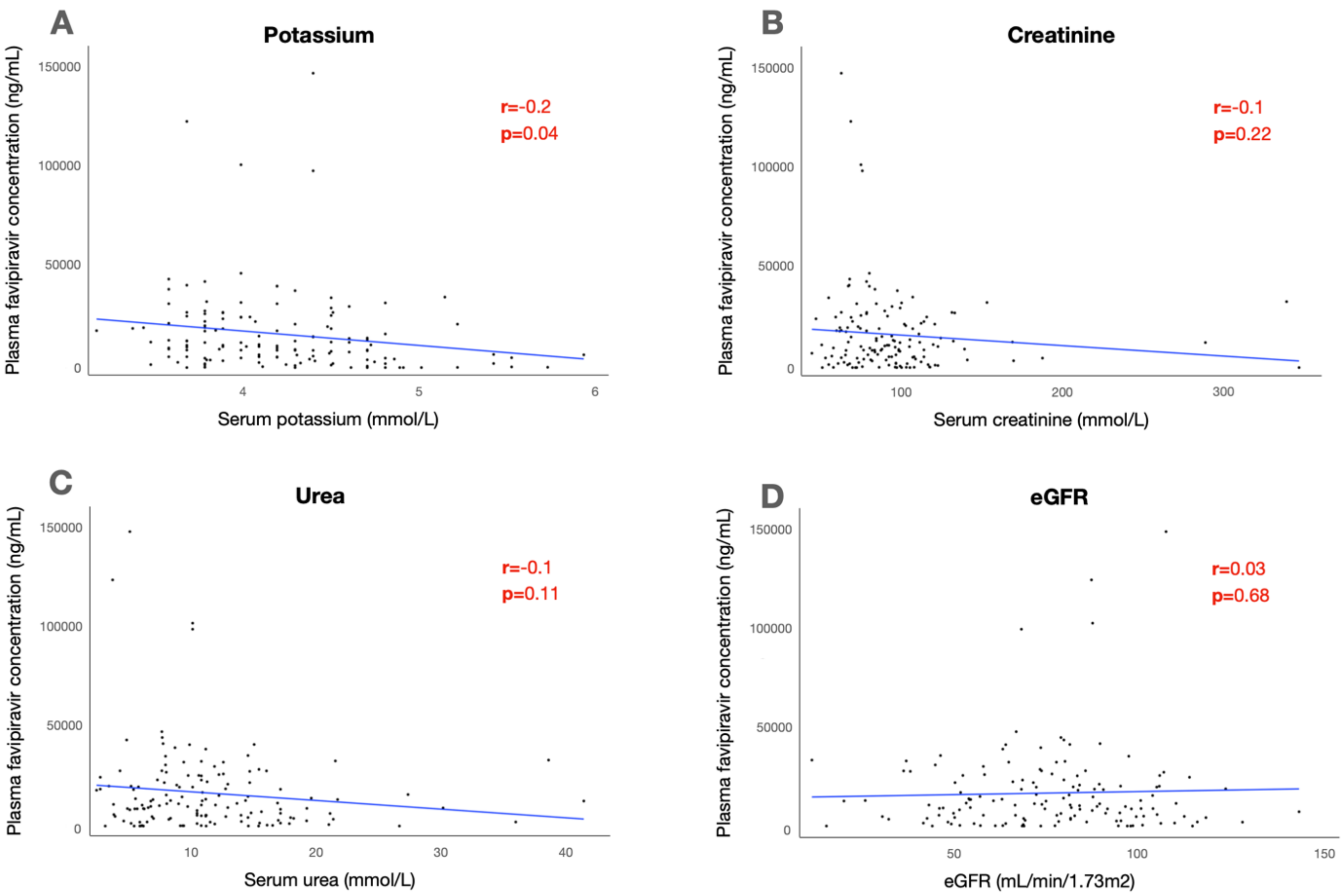

Supplementary Figure 3. Plasma favipiravir concentrations correlated with renal markers

Patient plasma favipiravir concentrations were quantified days 5-10 post treatment initiation and correlated against **A)** baseline serum potassium levels; **B)** baseline serum creatinine levels; **C)** baseline serum urea levels and **D)** eGFR. Pearson's correlation coefficient was calculated to assess the linear relationships between favipiravir concentration and renal markers.

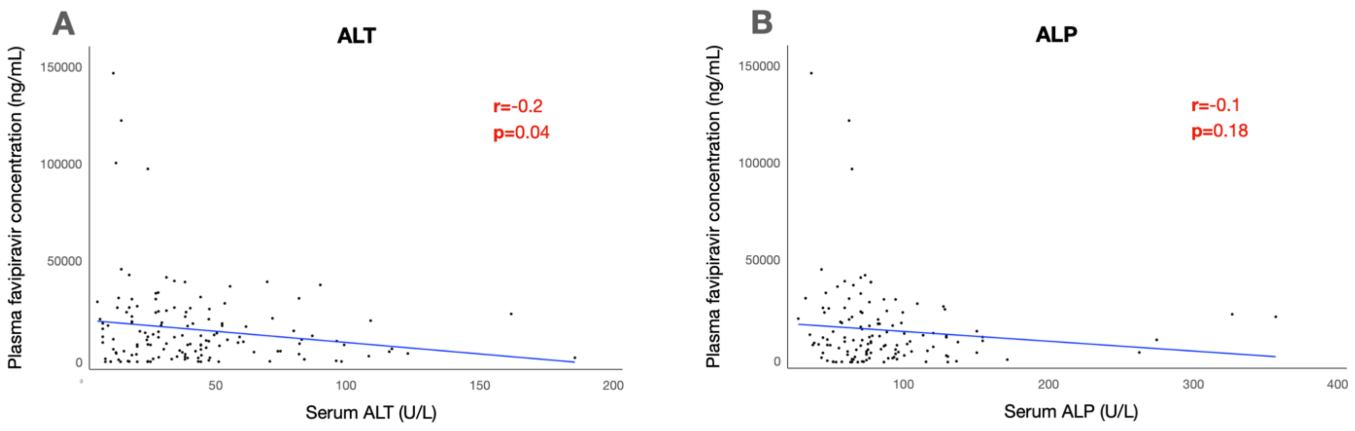

Supplementary Figure 4. Plasma favipiravir concentrations correlated with hepatic markers Patient plasma favipiravir concentrations were quantified days 5-10 post treatment initiation and correlated against **A)** baseline serum ALT levels and **B)** baseline serum ALP levels. Pearson's correlation coefficient was calculated to assess the linear relationships between favipiravir concentration and renal markers. (SI conversion factor: to convert U/L to µkat/L, multiply by 0.0167).

Supplementary Tables

Supplementary Table 1. Seven-category ordinal scale

Point	Description
**1**	Not hospitalised with resumption of normal activities
**2**	Not hospitalised, but unable to resume normal activities
**3**	Hospitalised, not requiring supplemental oxygen
**4**	Hospitalised, requiring supplemental oxygen
**5**	Hospitalised, requiring nasal high-flow oxygen therapy, non-invasive mechanical ventilation, or both
**6**	Hospitalised, requiring extra-corporal membrane oxygenation, invasive mechanical ventilation, or both
**7**	Death
Shah et al 2023 *The Lancet Respiratory Medicine*	

Supplementary Table 2. Post hoc pairwise chi-squared comparisons of BMI categories amongst patients who reached target plasma favipiravir concentrations compared to those who did not

Pairwise comparison	p value
Underweight (BMI <18.5) vs healthy weight (BMI 18.5-24.9)	1.0
Underweight (BMI <18.5) vs overweight (BMI 25.0-29.9)	0.46
Underweight (BMI <18.5) vs obese(BMI 30.0-39.9)	0.27
Underweight (BMI <18.5) vs severely obese (BMI >40)	0.14
Healthy weight (BMI 18.5-24.9) vs overweight (BMI 25.0-29.9)	0.17
Healthy weight (BMI 18.5-24.9) vs obese (BMI 30.0-39.9)	0.05
Healthy weight (BMI 18.5-24.9) vs severely obese (BMI >40)	0.08
Overweight (BMI 25.0-29.9) vs obese (BMI 30.0-39.9)	0.69
Overweight (BMI 25.0-29.9) vs severely obese (BMI >40)	0.33
Obese (BMI 30.0-39.9) vs severely obese (BMI >40)	0.52
Differences between groups were evaluated by pairwise chi-squared tests.	

Supplementary Table 3. Post-hoc pairwise comparisons of the 28 day survival of favipiravir recipients by BMI category

Pairwise comparison	p value
Healthy weight (BMI 18.5-24.9) vs overweight (BMI 25.0-29.9)	0.49
Healthy weight (BMI 18.5-24.9) vs obese (BMI 30.0-39.9)	0.32
Healthy weight (BMI 18.5-24.9) vs severely obese (BMI >40)	0.15
Overweight (BMI 25.0-29.9) vs obese (BMI 30.0-39.9)	0.73
Overweight (BMI 25.0-29.9) vs severely obese (BMI >40)	0.03
Obese (BMI 30.0-39.9) vs severely obese (BMI >40)	0.01
Pairwise differences between groups were evaluated by log-rank test	

Supplementary Table 4. Post-hoc pairwise comparisons of the 28 day survival all patients by BMI category

Pairwise comparison	p value
Healthy weight (BMI 18.5-24.9) vs overweight (BMI 25.0-29.9)	0.33
Healthy weight (BMI 18.5-24.9) vs obese (BMI 30.0-39.9)	0.33
Healthy weight (BMI 18.5-24.9) vs severely obese (BMI >40)	0.97
Overweight (BMI 25.0-29.9) vs obese (BMI 30.0-39.9)	0.97
Overweight (BMI 25.0-29.9) vs severely obese (BMI >40)	0.34
Obese (BMI 30.0-39.9) vs severely obese (BMI >40)	0.34
Pairwise differences between groups were evaluated by log-rank test	

Supplementary Table 5. Post hoc pairwise comparisons of the 28 day survival standard care recipients by BMI category

Pairwise comparison	p value
Healthy weight (BMI 18.5-24.9) vs overweight (BMI 25.0-29.9)	0.39
Healthy weight (BMI 18.5-24.9) vs obese (BMI 30.0-39.9)	0.39
Healthy weight (BMI 18.5-24.9) vs severely obese (BMI >40)	0.39
Overweight (BMI 25.0-29.9) vs obese (BMI 30.0-39.9)	0.76
Overweight (BMI 25.0-29.9) vs severely obese (BMI >40)	0.76
Obese (BMI 30.0-39.9) vs severely obese (BMI >40)	0.76
Pairwise differences between groups were evaluated by log-rank test	
